# New non-cognitive procedures for medical applicant selection: a qualitative analysis in one school

**DOI:** 10.1186/1472-6920-14-237

**Published:** 2014-11-07

**Authors:** Sara Katz, Shlomo Vinker

**Affiliations:** Research Department and English for Academic Purposes, Sha’anan Academic College, 7 Hayam Hatichon Street Kiriyat Shmuel, POB 906, Haifa, 2640007 Israel; The Department of Family Medicine, Sackler School of Medicine, Tel Aviv University, Tel Aviv, Israel

**Keywords:** Admissions, Medical school selection, Non-cognitive attributes, Qualitative methods, Holistic rubric

## Abstract

**Background:**

Recent data have called into question the reliability and predictive validity of standard admission procedures to medical schools. Eliciting non-cognitive attributes of medical school applicants using qualitative tools and methods has thus become a major challenge.

**Methods:**

299 applicants aged 18–25 formed the research group. A set of six research tools was developed in addition to the two existing ones. These included: a portfolio task, an intuitive task, a cognitive task, a personal task, an open self-efficacy questionnaire and field-notes. The criteria-based methodology design used constant comparative analysis and grounded theory techniques to produce a personal attributes profile per participant, scored on a 5-point scale holistic rubric. Qualitative validity of data gathering was checked by comparing the profiles elicited from the existing interview against the profiles elicited from the other tools, and by comparing two profiles of each of the applicants who handed in two portfolio tasks. Qualitative validity of data analysis was checked by comparing researcher results with those of an external rater (n =10). Differences between aggregated profile groups were checked by the Npar Wilcoxon Signed Ranks Test and by Spearman Rank Order Correlation Test. All subjects gave written informed consent to their participation. Privacy was protected by using code numbers.

**Results:**

A concept map of 12 personal attributes emerged, the core constructs of which were motivation, sociability and cognition. A personal profile was elicited. Inter-rater agreement was 83.3%. Differences between groups by aggregated profiles were found significant (p < .05, p < .01, p < .001).

A random sample of sixth year students (n = 12) underwent the same admission procedure as the research group. Rank order was different; and *arrogance* was a new construct elicited in the sixth year group.

**Conclusions:**

This study suggests a broadening of the methodology for selecting medical school applicants. This methodology differentiates between both individuals and groups, providing a personal attribute profile of applicants, useful for admission procedures. The qualitative procedures are cost-effective, can easily be taught and used by faculty members. The predictive validity of the presented model requires a longitudinal trial.

## Background

Most formal medical school admission systems tend to primarily assess academic achievement in science domains and cognitive abilities [1–5]. In Israel the psychometric entrance Test (PET) is a standardized test administered and weighed for university, college and medical school admissions. A large body of research demonstrated the high predictive ability of the PET [6]. The test ranks all applicants on a uniform scale and, compared to other admission tools, is less affected by differences in applicants’ backgrounds or other subjective factors. In general, students who receive high PET scores are more successful in their academic studies than students who receive low scores.

Medical school selection has traditionally also been based on interviews, personal statements and evidence of practical experience to assess non-cognitive factors such as oral communication skills, motivation and suitability for a career in medicine [7]. Recent work has encouraged admission committees to consider standardized non-cognitive attributes as well as academic performance [8].

The present study investigates an admission system model that used the PET, an exclusion interview and a “resume essay” for accepting applicants to medical school. After having achieved a certain score in the PET that allows acceptance to medical school, applicants underwent a mandatory exclusion interview, designed to prevent entry of presumably psychologically unsuitable candidates. The exclusion interview has been used in the admission system for more than twenty years. A brief “resume essay”, written by the applicant while waiting, was given to the interviewers before beginning interviewing. Faculty members conducting this admission process admitted that they knew very little about the applicants, and that the existing tools supplied insufficient information in that regard. While the PET is generally able to predict academic success, there may be a number of examinees who do not do well on the test but nonetheless succeed in their studies, and vice versa. Neither is the test a direct measure of such factors as motivation, creativity, and diligence, which are definitely related to academic success – although some of these elements are measured indirectly.

Recent data have called into question the reliability and predictive validity of standard admission procedures to medical schools [9–11]. High academic scores are insufficient for being a good physician [12]. Therefore, a more holistic approach to selection – taking into consideration non-cognitive attributes – needs to be developed and applied [13]. Medical schools are increasingly including non-cognitive attributes (NCA’s) in addition to cognitive ability in the admission process [11, 14–16]. Reliably eliciting personal attributes in applicants has thus become a major challenge of this study.

Recent advances in social sciences have shifted the psychometric approach to a qualitative naturalistic one, emphasizing personal attributes, such as self-efficacy, which is a component of motivation, in addition to cognitive skills, as determinants of performance [17, 18]. Self-efficacy (SE) refers to the belief in one’s capability to organize and execute the courses of action required to produce given attainments [19]. Self efficacious people are those who believe they have the power to achieve results, and act accordingly. SE influences the choice of activities, thought processes, and affective states, and regulates motivation [19].

We view our future physician as an expert who will possess medical, clinical and procedural skills as well as professional attitudes. He will have to collect and interpret information, and make decisions. He should be able to effectively communicate with patients, health providers and community, and effectively collaborate within a team. We would like to have a lifelong reflective learner who will attain high personal behavior standards. To that end, there is a need to explore ways of eliciting a variety of personal attributes. The question that arises is how can we elicit medical school applicants’ NCA’s in addition to their cognitive ones?

The aim of the current study is to attempt to use new methods to select medical school applicants. In order to elicit tacit knowledge on the applicant’s personality, new procedures that are based on qualitative approaches are added to the existing ones. These procedures are based on positive perspectives embedding in them current assumptions of assessment [17]. Creating a comfortable setting would increase openness under which negative components of personality as well as positive ones would emerge.

A contextual qualitative approach utilizing a variety of strategies and tools, such as portfolios, reflection, open interviews, open questionnaires, field notes, observations, and documents, assess NCA’s [17, 20]. This study emphasizes a shift from generating scores to generating profiles. Underlying concepts here include: *intelligence*, defined as multidimensional and non-fixed [21]; *thinking*, including meta-cognition and tacit knowledge, and *mental processes* that interact significantly in the socio-cultural context. It would appear that there is no single best way to assess NCA’s [17].

## Methods

### Subjects

**Figure 1:**
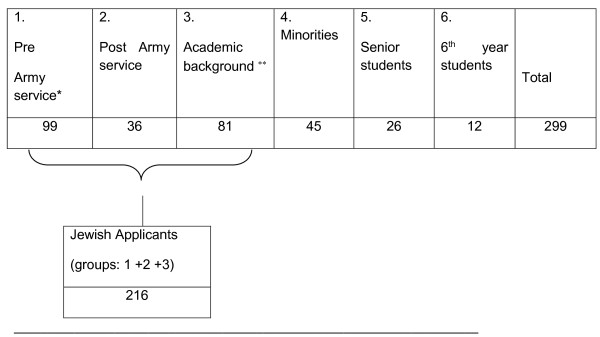
**Number of applicants in groups of biographical background.**

299 participants aged 18–26 years, from a northern Israeli medical school, took part in the present study. 261 were medical school applicants, and 38 senior medical students. Applicants differed according to demographic background: pre (n = 99) and post (n = 36) military/national service, applicants with academic background (n = 81), (are applicants with a university degree obtained before application to medical school), Jewish medical applicants, ethnic minorities (Moslems, Christians, Druze and others, n = 45), and according to acceptance status: accepted, rejected, applicants who were not accepted or rejected yet but had positive interviews, applicants who were not accepted or rejected yet but had negative interviews, applicants who were accepted and have started to study, those who were accepted and left, those whose interviews were positive, but were rejected due to a decision to cut back on student numbers, those who were rejected and their interviews were negative, males and females (See Figure 1 and Table 1).

**Table 1:** **Number of applicants in groups according to acceptance status** *

1. Accepted	2. Rejected	3. Male	4. Female	5. Positive Interview	6. Negative Interview	7. Accepted & Study	8. Accepted & Left**	9. Rejected & Positive Interview^***^	10. Rejected & Negative Interview
116	145	143	118	226	35	94	22	112	35

*Groups created out of the pool of applicants (n = 299). One applicant can be a member of more than one group at the same time.

**Applicants who left out of choice or were placed by the army in another Medical School.

***Applicants with lower psychometric grades than others whose interview scores were positive.

### Design

This is a qualitative case study that uses qualitative tools and methodology. These methods consist of systematic, yet flexible guidelines for collecting and analyzing data to construct abstractions. The flexibility and the openness of the qualitative approach enable revealing tacit knowledge [22].

### Research tools

Six research tools were developed for this study in addition to the two existing ones (an exclusion interview and a resume). These included:

1. **A Portfolio task:** Three days before the interview, the applicant was asked to prepare a written narrative, describing between 4–10 experiences of success in his life, providing evidence supporting the narrative. Three reflective questions were included:

- What does this story tell asbout your personality?
- What is its relevance to studying medicine?
- Why did you want us to read about it?

The objective was to reveal as much tacit knowledge as possible [23].

2. **An intuitive task:** applicants were asked to list the personal attributes of a good physician on a blank paper.
3. **A cognitive task**: applicants were asked to rank the attributes they had written from most to least important, and write field notes and explanations wherever they wanted.
4. **A personal task:** applicants were asked to list the good physician attributes they possessed.
5. **A Self-efficacy questionnaire** containing 36 statements on self-efficacy relevant to becoming a physician, which was constructed on the basis of a formal analysis of the self-efficacy (SE) component in literature [19], expert consultation, adaptation of Zimmerman and Bandura’s SE questionnaire [24], Prochaska’s SE questionnaire on health regulation [15], and field experience. Applicants were asked to write comments arising from the statements in the questionnaire, including thoughts, feelings and experiences evoked by the statements. The applicants’ reactions were analyzed qualitatively.
6. **Field notes** were taken by the researcher regarding the applicant’s behavior and comments during the interaction.

In addition, interviewers were encouraged to write as many comments as possible in addition to providing interview scores. Such comments were taken into account in the qualitative analysis, as was the “resume essay”.

### Procedure and data analysis

Participants were told that the study was independent of, and would have no effect on, the on-going admission process. All participants (n =300), except one gave written informed consent to their participation. The applicants were informed about the aims and methods of the study. They volunteered to participate in the hope of benefiting future applicants. Privacy was protected by using code numbers. Applicants were offered the opportunity to read the manuscript. No applicant can be identified in the manuscript in any way. The manuscript does not contain any specific identifying information on any individual or group.

The applicants handed in their portfolio and wrote the resume before the interview started. They were interviewed by teams consisted of two faculty members, one of whom was a psychiatrist. After the interview they met with the researcher to openly discuss or talk about their portfolio or about their interview. Then they answered the questions of the other tools. The applicants could get the results of the analysis if they wished to. The evidence they handed in with the portfolio task included very creative and interesting objects such as, cakes, delicate works of art, drawings, photographs, presents they got, letters and certificates. The objects they brought were given back to them when the analysis was finished.

The qualitative methodological frame used for analysis was the criteria oriented methodology, which assumes that open analyses are often influenced by perspectives and views that researchers hold [25, 26]. Charmaz (2006) argues that preconceived theoretical concepts may provide starting points for looking *at* the data but they do not offer automatic codes *for* analyzing these data [27]. Of all qualitative frames, this one is the closest to the quantitative methodology.

An applicant profile of personal attributes was generated using constant comparative analysis [26, 28] and the grounded theory techniques [29]. The unit of analysis was an idea or an object. The units were coded into categories through three phase coding: the initial, the axial and the selective coding [30–32]. Each unit was compared with other units or with properties of a category. Analyses began during data collection and continued after its conclusion. Recurring themes were examined, gathered under criteria and the criteria were gathered under categories (e.g. The category of “reasons for studying Medicine” contained two criteria: “external reasons” and “internal reasons”) (See also Table 2). Under the restrictive qualitative rules of the constant comparison analysis methodology, core constructs were formed. A core construct is a category which contains dense descriptions of evidence supporting it. The theoretical coding stage was the sophisticated level of coding that followed the three phase coding. It specified possible relationships between the categories that had been developed before [26, 27, 33]. The constant comparison of units was adapted, changed, redesigned as the study proceeded, and resulted in a refined list of categories that were developed into conceptual abstractions called constructs [34]. The concept map, was sampled only when repetition of the same constructs was obtained from multiple cases, and when new units did not point to any new aspect. Then the list of constructs became theoretically saturated [32].

**Table 2:** **Criteria and representative quotation examples**

**“Perseverance”**	- As illustrated in the picture, with bandaged wounded fingers I go on playing. When you become stronger the music becomes purer.
**“Interest in people”**	- I stayed in that school in India seven months. I studied these children, I even painted their school.
**“Poor social cooperation”**	- I couldn’t get along with my peer. I left the job.
**“Poor Affect regulation”**	- Let me out for just a minute, I need to smoke a cigarette, I feel I feel so bad, I’m sure they wanted to fail me.
	- Sorry, it’s against regulations here.
	- But I have to … (leaves the room). (From a discussion with the researcher following the interview).
**“Empathy”**	- Lucky to be born in a happy, healthy world of opportunities, I’d like to help the needy. I stayed three days with that sick old man and took care of his leg.

The researchers stayed in the setting over time thus enabling interpretation of the meaning in individuals’ lives [34].

This methodology produced a profile of personal attributes for each subject. The profiles were scored on a 5-point scale holistic rubric (See Table 3). This efficient assessment device consisted of one scale where each dimension is related to at each point of the scale. It gives an overall description of what is expected at each level [17].

**Table 3:** **Holistic rubric for assessing an applicant profile**

**Dimension**	% of evidence in category	
**Criteria**		
	* A profile containing >14.29% evidence in any category will have 1 point (in the 5 point scale rubric). If the category is negative it will have a minus 1 point.	
	* A profile containing between 10% - 14.28% evidence in any category will have partial points according to the following description:	
	**% Evidence in Category**	**Scale Points**
	14.00 – 14.49	.9
	13.5 – 13.99	.8
	13.00 – 13.49	.7
	12.5 – 12.99	.6
	12.00 – 12.49	.5
	11.5 – 11.99	.4
	11.00 – 11.49	.3
	10.5 – 10.99	.2
	10.00 – 10.49	.1
**Benchmarks:**	A profile will score **1** if it contains >14.29% evidence in any category or has accumulated partial points according to the description on the table above.	
	A profile will score **2** if it contains >14.29% evidence in 2 categories or has accumulated partial points according to the description on the table above.	
	A profile will score **3** if it contains >14.29% evidence in 3 categories or has accumulated partial points according to the description on the table above.	
	A profile will score **4** if it contains >14.29% evidence in 4 categories or has accumulated partial points according to the description on the table above.	
	A profile will score **5** if it contains >14.29% evidence in 5 categories or has accumulated partial points according to the description on the table above.	

Note 1: The rubric was built according to a theoretical profile containing the 7 positive attributes that were elicited from the data. 100%: 7 (categories) =14.29.

Note 2: The result of the analysis is a list of themes classified in constructs. Then the numbers and the percentages of the themes on every construct are calculated and demonstrated by a pie. This constitutes an applicant profile.

Note 3: The words written in boldface letters are subtitles and score numbers.

Note 4: “*” is a symbol for a scoring criterion.

Qualitative validity of data gathering was checked according to the qualitative rules, by comparing the profiles elicited from the existing exclusion interview against the profiles elicited from all other tools [33], and by comparing two profiles of each of the applicants who handed in two portfolio tasks. Qualitative validity of data analysis was checked by comparing researcher results with those of an external rater (n =10).

This manuscript reporting adheres to RATS guidelines for reporting qualitative studies.

In order to gain additional insight into our qualitative analysis, we performed some quantitative checks. The profiles were aggregated into groups according to demographic differences and admission status, and the differences between them were checked by the Npar Wilcoxon Signed Ranks Test and by Spearman Rank Order Correlation Test.

Another group consisting of senior medical students in the School of Medicine was studied (n = 26). Their teachers were asked to divide them into two groups: one (n = 13) was considered “Good” from the NCA perspective by at least two teachers and the other (n = 13) was considered “Poor” from the same perspective. Protocols of these students’ original admission process (the interview and the short resume) were analyzed by the same methods used in the research group. Personal profiles were elicited and aggregated into two groups. Differences were checked by the Npar Wilcoxon Signed Ranks Test, and the Spearman Rank Order Correlation Test.

A random sample of 6^th^ year students (n = 12) went through the same admission procedure as the research group had done, but without the interview. Personal profiles were elicited and aggregated into one group, and then the differences between this group and the research group were checked by the Npar Wilcoxon Signed Ranks Test, and the Spearman Rank Order Correlation Test.

### Ethics

We declare that a prior ethical approval for conduct of this study was obtained from the research ethics review board of Shaanan College Ethics Committee (http://www.Shaanan.ac.il).

## Results

A qualitative analysis of the data generated a concept map of 17 constructs; 12 personality constructs and five position constructs. The latter consisted of positions towards the interview, the interviewers, the portfolio and the questionnaires.

The 12 personality constructs were generated out of a large data set of 47,251 units of evidence. The units were classified into 95 criteria that were later classified into categories. These categories formed the 12 constructs of the concept map. Seven personality constructs were positive and five were negative.

The seven positive personality construct attributes were:

1. *Cognitive competencies* such as problem solving, formulating questions, searching for relevant information, efficient use of information, conducting observations, investigating, inventing and creating new theses, analyzing data, oral and written expression,
2. *Meta-cognitive competencies* such as self-reflection, self-evaluation, capability of handling ethical problems in medicine, abstract thinking.
3. *Affect* including empathy and sensitivity to human needs.
4. *Meta-affective competencies* including coping with frustrating and stressful situations, functioning under ambiguity.
5. *Motivation* including curiosity, interest in people, self-efficacy, responsibility.
6. *Social competencies* such as leading discussions, leading people, persuading, cooperating, working in groups.
7. *Motor skills* such as drawing, playing music, sports, dancing, skiing, hiking.

The five negative personality construct attributes were:

8. *Poor cognition*
9. *Immaturity*
10. *Poor affect*
11. *Psychopathology*
12. *External motives.*

### Emergent findings

1. *Motivation* was the dominant core construct containing the densest descriptions of evidence (26.3% of all applicants’ units belonged to the category of *Motivation*). *Sociability* and *cognition* were second and third (19.8%, 19.5%). The participants invested much effort in collecting evidence showing high motivation to study medicine.
2. A unique personal attribute profile for each subject emerged showing a divergent pool of personal attribute profiles and fine levels of differentiation between applicants. For example, two profiles of applicants of similar demographic background, both of whom were accepted by the standard procedure and both having the same scores on the exclusion interview, (3.9 on a 1–5 point scale), differed. The first applicant’s category of *Cognition* contained 24% out of the total amount of the units he had, while the second student’s category of *Cognition* contained 8.5% out of the total amount of the units he had. The first applicant’s category of *Meta-cognition* contained 23% out of the total amount of the units he had, while the second student’s category of *Meta-cognition* contained 14% out of the total amount of the units he had. The first applicant was cognitively and meta-cognitively more skillful than the other but socially less skillful (19% <33%) than the other. The Qualitative analyses showed delicate (precise) differences between the profiles.
3. Qualitative validity of data gathering was checked by comparing the profiles elicited from the exclusion interview, against the profiles elicited from the new tools. The profiles were assessed by a holistic rubric scored on a 5-point scale (Table 3). These scores corresponded with the exclusion interview scores, r = .711, p < .01 (1-tailed), high correlation, indicating the extent to which data gathering was a true description of reality. Of note, six applicants handed in a second portfolio task, claiming dissatisfaction with their first effort. The two portfolios from the same participant were compared and emerged as identical. The second portfolios actually had more evidence, yet the percentages of the criteria in the categories of their profiles did not change.

Ten cases were randomly chosen for analysis by an external rater. Agreement between the raters was of 83.3%. Five disagreements were as close to agreement as -1, +1, which brings about a total agreement of almost 87.5%. Experience showed that our procedure of profiles elicitation was easily taught and applied by faculty members.

4. Aggregation of data into groups of similar demographic background and applicant status generated 15 groups of applicants. We checked the differences between the aggregated profiles using the Npar Test Wilcoxon Signed Ranks Tests.

**Figure 2:**
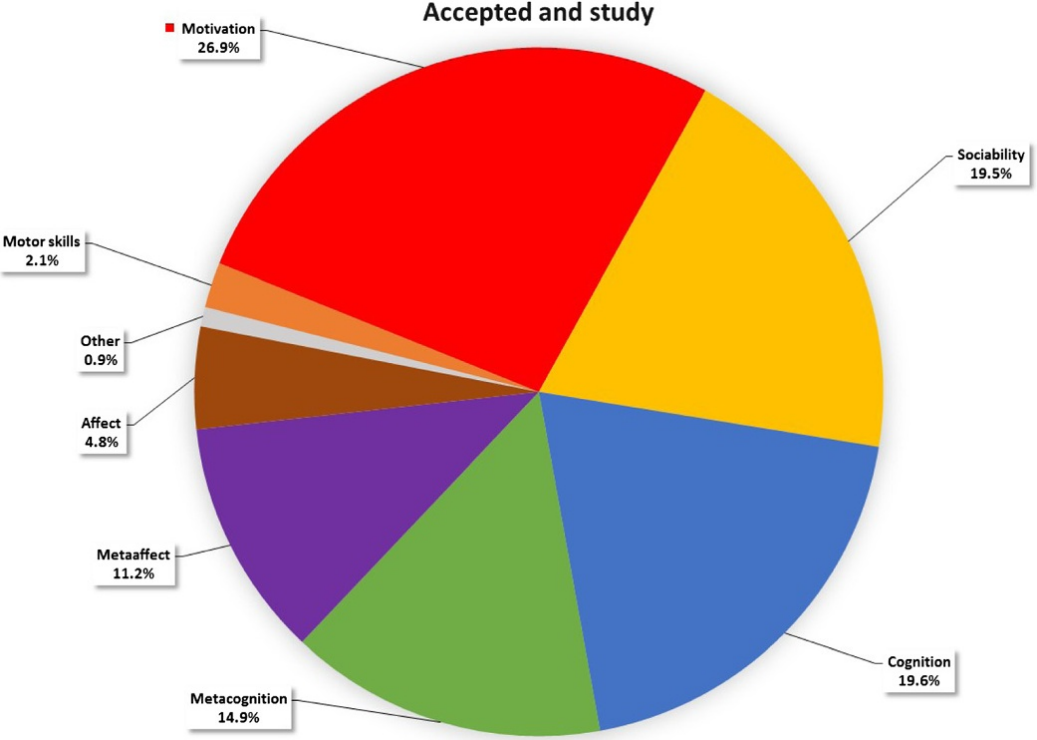
**Group profile of applicants who were accepted and began studies.**

**Figure 3:**
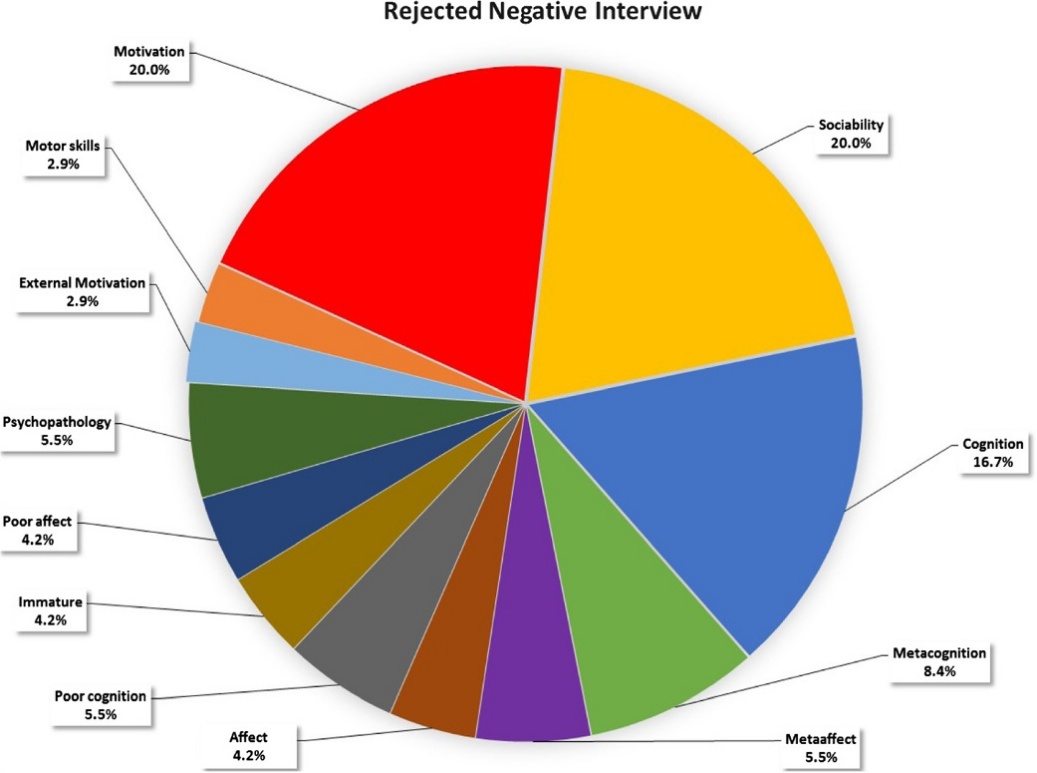
**Group profile of applicants who were rejected with negative interview.**

Significant differences were found in 12 out of 13 Npar Test Wilcoxon Signed Ranks Tests (Table 4). An illustration of the *Accepted + began studies* and R*ejected + negative interview* difference is shown in Figures 2 and 3. The former had higher scores in meta-cognitive (14.9% >8.4%) and meta-affective attributes (11.2% >6%) and less negative attributes (1% <23.5%) in the group profile than the latter (M per person in a category: 16 > 8)^a^.

**Table 4:** **Npar Wilcoxon signed rank Tests, Z values for two related samples**

	Rejected negative interview	Negative interview	Minorities	Female	Post army service	Academic background	Rejected	Accepted & study
Accepted & study	a							
	-1.883*							
Positive interview		b						l
		-1.961*						-2.667**
Rejected & positive interview	c							
	-1.883*							
Jewish applicants:			d					
Pre + Post service, + Academic Background			-2.746**					
Male				e				
				**-2.981				
Pre army service			f		h	i		
			-2.353**		***-3.059	**-2.353		
Academic backgroung			g		j			
			-2.040*		***-3.059			
Accepted							k	
							*** -3.059	
Post army service			- .863					

p < .05 (1-tailed) *, p < .01 (1-tailed) **, p < .001 (1-tailed) ***.

a – l = number of comparisons.

- The “Good and the “Poor” group profiles (total n = 26) were analyzed using the same qualitative research method based on the two pre-existing tools (the exclusionary interview and the short resume). The results were aggregated for the two groups. The “Good and the “Poor” aggregated group profiles were compared. Both groups’ results showed a very high correlation: r = .96, p < .01 (2- tailed), with no significant difference found in the Npar Wilcoxon Signed Ranks Test between the groups: z = -.679, p < .25 (1-tailed). A sufficiently detailed database to differentiate between the group profiles using the pre-existing tools could not be generated; further development was needed in this area.
- A random sample of 6^th^ year students (n = 12) went through the same admission procedure as the research group had done, only without the interview. The interview data was thus removed from the research group as well. The qualitative analysis of data revealed personal attribute profiles of the 6^th^ year students that were aggregated into a group profile. The difference between the research group and the student group profiles was checked:
- Difference in the Npar Wilcoxon Signed Ranks Test was significant, z = -3.059, p < .001 (1-tailed).
- Applicants had collected more units of evidence, than did the 6^th^ year students (M per person in a category: 15 > 4; average size of portfolio: 30 > 1-2 pages).
- Rank order was different: *motivation* was the first category for the applicants and twice as large (27% >13%), but only the fifth category for the students. The first category of the students was *cognition,* but it was only the second for the applicants.
- *Affect* was almost four times greater for the students than the applicants (18% >4.6%).
- Interestingly, *meta-affect* was twice as large for the applicants as it was for the students (10.7% >4.5%),
- The qualitative analysis elicited a new construct specific to the 6^th^ year students’ group: arrogance, showing off, indifference, excessive self-esteem, and criticism of hospital arrangements, staff, or tutors.

## Discussion

### A paradigm shift for assessing applicants

This study suggests a broadening of the methodology for selecting medical school applicants. Kulatunga and Norman [35] have shown that traditional academic predictors, Grade Point Average (GPA), and Medical College Admission Test (MCAT) have most utility in predicting future academic and licensing examination performance. However, the relationship between GPA and clinical performance is less clear. Much of the variance in academic performance remains unexplained. Other variables, perhaps non-cognitive ones, may contribute to both academic and clinical performance outcomes, and the literature [10, 11] offers little guidance on how best to assess such characteristics.

There is limited evidence that the non-cognitive measures currently in use, such as autobiographical submissions, interviews, simulations and psychological inventories are in fact sufficiently reliable and valid to predict success [10, 11]. The high inter-rater reliability in simulations and interviews is found since all raters see the same performances [12]. However attempting to achieve validity through quantitative analyses that isolate components from their natural context is problematic, since traits are stable qualities with a high probability of occurrence in an almost infinite number of different contexts [12, 14, 36]. Thus, a holistic contextual perception that identifies personal traits, in a variety of settings at a specific period appears more effective for this purpose.

The qualitative paradigm uses a variety of authentic contexts, offers a fair chance for the applicants to present themselves as they wish, and a qualitative analysis method, that best suits this purpose. The applicants responded positively and were willing to energetically collect and present evidence on their achievements, actions, personality and experiences, revealing themselves without hesitation. The constructs were generated by repeated analyses, and validity was achieved by using an external rater. Under restricting qualitative rules, the long course of analysis, reiterations, turning to literature and back to the field, refining the analyses, all while collecting the data, subsequently generated the concept map [22, 34].

### Eliciting tacit knowledge

A twelve-construct concept map has emerged here. Since the admission process is typically very competitive with more applicants than available slots, the main concern of applicants was unsurprisingly *motivation*. Applicants spent their greatest efforts showing how much they wanted to become physicians, emphasizing their cognitive and social competences. These efforts reflected the applicants’ current perceptions on attributes of a desired physician, but the openness of the tools clearly brought out negative personal attributes as well. One of the participants for example, has spent his whole portfolio assignment showing certificates and high grades. His profile scored very low (1 on the 1–5 point scale rubric), as he had very little of all other competencies.

### Eliciting a detailed personal profile

The most important finding was a detailed personal profile of attributes, with percentages that reflected the individual strength in each. We possess a profile pool reflecting 299 different ‘personalities’. These very detailed profiles show fine differentiations among applicants. Admission committees and faculty members may find this extremely useful in the decision-making process of selecting applicants with desired attributes.

Is there an ideal profile? Selected candidates must be those best suited to the study of medicine and most likely to become competent physicians. This issue has been dealt with in medical schools all over the world [12]. Given that profiles of good candidates may differ, the question is which one should be selected; perhaps several differing profiles should be the norm? The cost of selecting inappropriate applicants is substantial for both the faculty and society. We may consider a profile rating as high if it includes the maximum number of the seven positive core constructs. The profile enables selection according to faculty perceptions and goals, empowering positive attributes, or changing negative ones through education. This requires a discourse to be generated in faculties. The long-term predictive power of these profiles remains a major research challenge.

### SE – the best motivational component to predict performance

The literature to date pertaining applicants wishing to study Medicine has not placed much focus on SE as a factor to be considered in the decision making process by medical faculties [20]. Efficacy beliefs operate as a key factor in the generative system of human competence, and are an important contributor to performance accomplishments, whatever the underlying skills might be. People need firm beliefs in their personal efficacy to turn concerns into effective actions. SE affects how well people manage requirements and challenges of their occupational pursuits. Specific personal preferences revealed through the SE questionnaire, illuminated/refined the emerging personal profile. The evidence from this study supports placing emphasis on SE as an important factor in decisions pertaining to applicant acceptance.

### Discrimination between groups of applicants

The significant difference generated between group profiles demonstrates the ability to elicit typical similarities of various groups. Applicants who were *accepted* and had *positive interviews,* showed higher motivation, spent more effort and had less negative personal attributes than those who were *rejected* or had *negative interviews* (See Figures 2 and 3)*.*

Jewish applicants scored higher in effort and motivation and had less negative attribute scores than *ethnic minorities*. The differences may be explained by cultural, environmental and socioeconomic differences between Jewish applicants and *Ethnic Minorities* [37, 38] e.g. army service^a^, academic backgrounds or school environments. However, linguistic and cultural limitations of the researchers may constrain effective data gathering from those with differing backgrounds and this area requires further study. G*ender* groups unsurprisingly showed no other difference except that women invested and spent more efforts than men.

Significant differences found between the 6^th^ year students and the research group is important. The 6^th^ year students did not need to demonstrate *motivation*, which thus ranked fifth in their data. However, they gathered evidence for cognitive competencies thus demonstrating that their acceptance was a success. The high rank order of *affect* in their profile suggests that affect was being successfully nurtured in their studies. For the applicants, on the other hand, *motivation* is the most important issue, they spent much effort in gathering evidence, and prepared longer portfolios than the 6^th^ year students did. *Meta-affect* was twice as large for the applicants as it was for the 6^th^ year students, which might hint at slightly unrealistic appraisal of the inexperienced applicants. Most striking, a new construct specific to the 6^th^ year group emerged: *arrogance* – i.e. showing off, indifference, excessive self-esteem, and criticism of hospital arrangements, staff, or tutors, which deserves further study.

### Limitations of the study

Due to technical problems, the post-army service group who went through the admission process during March- June were not included in the study. Many of them were accepted. Therefore, in this study, this group is small and not typical. This is probably the reason for not finding a significant difference between this group and the *minorities*.

### The exclusion interview function

Correlation between the exclusion interview, and the other tools (r = .71) shows that the exclusion interview successfully identifies problem cases, but does not supply a sufficiently detailed database to discriminate between individuals, and thus cannot predict future performance. Therefore, while the exclusion interview effectively does what it sets out to do, the need for a new set of discriminatory tools is clearly demonstrated in this study.

### Eliciting and measuring constructs

There is a general agreement in the literature that the admission process should include assessment of both cognitive and non-cognitive characteristics of applicants [11, 12, 14, 23, 39]. Grades (i.e. past academic performance) can easily be assessed but measurement of non-cognitive attributes is much more difficult. Grades are an index of intelligence and motivation, as well as mastery of subject areas and remain the best predictor of future performance [5, 10, 11, 40]. However, developing better measures of other characteristics that are equally important but measured with difficulty [41] remains a priority, and this study is an attempt on this direction.

## Conclusions

This study addressed the eliciting of basic NCA’s in medical school admission. This procedure might take a few months work for two faculty members, which is cheap. An easily applicable method of evaluation that uses multiple observations across contexts to provide an accurate picture of constructs and measure them was developed. All aspects of qualitative validity were checked, and the results are able to contribute to the selection process. Eliciting NCA’s may represent an innovative approach to the formal measurement of personal and interpersonal skills of applicants, and show promise as complements to cognitive examination components in medical school admission processes. But more research is needed to establish its predictive validity.

As a next step, an analytic rubric for assessing students’ performance needs to be constructed and subsequently compared to the applicant’s profiles. This procedure requires a longitudinal trial. An admission process that provides a thorough, fair, reliable and valid cost-effective assessment of applicants remains an important goal for all medical education programs.

## Endnote

^a^More illustrations of detailed data is available by request.

## Authors’ information

Sara Katz is an educational psychology researcher. Her main is in self-efficacy self-regulation, instruction and assessment. She is the author of three books and has published and presented papers on these subjects.

## Abbreviations

PET: Psychometric entrance test
SE: Self-efficacy
NCA’s: Non cognitive attributes.
